# DAILY CONTEXTS AND HEALTH OUTCOMES ACROSS ADULTHOOD

**DOI:** 10.1093/geroni/igac059.1024

**Published:** 2022-12-20

**Authors:** Joanna Hong, Meng Huo, David Almeida

## Abstract

Different daily contexts (i.e., social, behavioral, emotional) influence psychological and physical health. In particular, daily contexts change across adulthood and identifying salutary daily contexts is vital for improving the health of middle-aged and older adults. This symposium adds to a burgeoning literature and presents studies that examine how different daily contexts (social - friend interactions, behavioral - electronic usage, emotional - affective response) impact health outcomes across adulthood. Ng et al. found that friend interactions were associated with poorer heart rate variability throughout the day among Black, but not White adults. Kim et al. showed that evening computer use, a health-impairing behavior, is associated with greater sleep disturbances on the following nights among older adults. Rush et al. found that low and high levels of negative affect reactivity to stressors (increased negative affect in response to stress) were associated with poorer health. Yet, moderate levels of stress reactivity predicted better health. Similarly, Hong et al. found that less positive affect reactivity to stressors (decreased positive affect in response to stress) was protective against elevated systolic blood pressure across stressful days. Lastly, Huo et al. identified an association between older adults’ empathy and daily rumination (i.e., repetitive thoughts) over distress, but observed that the link was attenuated on days when older adults maintained more stable levels of negative affect. Together, findings may inform future interventions aimed at increasing health across adulthood. Dr. Almeida will serve as the Discussant and summarize these studies with regard to their theoretical and methodological contributions.

